# Ultrasound-measured cutaneous-epiglottic distance for predicting difficult laryngoscopy: an observational study

**DOI:** 10.1016/j.bjane.2025.844637

**Published:** 2025-05-09

**Authors:** Luis Henrique Cangiani, Rodrigo Leal Alves, Glenio B. Mizubuti, Rodrigo Moreira e Lima, Lais Helena Navarro e Lima

**Affiliations:** a Clínica Campinense de Anestesiologia, Fundação Centro Médico Campinas, Campinas, SP, Brazil; b Hospital São Rafael, Salvador, BA, Brazil; c Universidade Estadual Paulista (UNESP), Departamento de Anestesiologia e Especialidades Cirúrgicas, Botucatu, SP, Brazil; d Queen’s University, Department of Anesthesiology and Perioperative Medicine, Kingston, Canada; e University of Manitoba, Department of Anesthesiology, Perioperative, and Pain Medicine, Winnipeg, Canada

**Keywords:** Airway management, Endotracheal intubation, Epiglottis, Laryngoscopy, Ultrasonography

## Abstract

**Background:**

Ultrasound (US) allows for rapid bedside airway assessment. We aimed to evaluate the US-measured cutaneous-epiglottic distance (CED) in predicting difficult laryngoscopy (Cormack-Lehane grades 3‒4). We also evaluated the potential association between CED, sex, patient’s body mass index (BMI), and the independent associations between CED and increased odds for Cormack-Lehane grades 3‒4 (secondary outcomes).

**Methods:**

Patients aged 18‒70 years scheduled for elective surgeries under general anesthesia with tracheal intubation were included. Those with a BMI > 35 kg.m^-2^ and/or previous history of difficult intubation were excluded. CED was measured with patients anesthetized before tracheal intubation. Age, sex, BMI, type of surgery, and number of attempts until successful tracheal intubation were recorded. Receiver operator characteristic (ROC) curve analysis was performed to evaluate CED’s clinical relevance. Secondary analyses compared the association between CED and BMI in patients with Cormack-Lehane grades 1‒2 versus those with grades 3‒4. The relationship between CED and BMI was assessed using multiple linear regression. Binary logistic regression was employed for predicting Cormack-Lehane grades 3‒4 as a dichotomous outcome with CED and BMI as a covariate.

**Results:**

ROC curve analysis revealed an area under the curve of 0.899 (p < 0.001). The maximum CED cut-off point (by Youden index) was 25.6 mm. CED and BMI were positively correlated, and both were independently associated with an increased odds for difficult laryngoscopy [odds ratio for CED = 1.81, 95% confidence interval (CI) 1.35‒2.41; BMI = 1.30, 95% CI 1.05‒1.59].

**Conclusion:**

US-measured CED has a high discriminatory capability for predicting lower (1‒2) and higher (3‒4) Cormack-Lehane grades during direct laryngoscopy. CED was positively correlated with BMI and was independently associated with higher odds for difficult laryngoscopy.

## Introduction

Critical and potentially fatal situations, such as ‘cannot intubate, cannot oxygenate,’ are estimated to occur in one of every 1000 elective surgeries and in one of every 250 rapid sequence intubations.^1^ Proper preoperative evaluation can help manage a difficult airway; therefore, preoperative airway assessment is paramount for reducing potentially related risks.

Bedside tests and measurements, such as the Mallampati classification, thyromental distance, sternomental distance, neck circumference, and interincisor distance are routinely used in clinical practice to predict difficult tracheal intubation.^2^^,^^3^ However, when used independently, most standard tests have limited predictive value, particularly in patients with no evidence of increased risk for difficult airway, in whom challenging situations may occur.^2^^,^^4^^,^^5^ Concerning the easiness of airway manipulation, according to the Cormack-Lehane visualization classification, patients with Cormack-Lehane grades 1 or 2 are considered easy to intubate, while those with grades 3 or 4 are considered difficult.^1^^,^^6^ However, this classification is only possible with direct visualization during laryngoscopy. Therefore, an index that can accurately predict difficult intubation scenarios *prior* to laryngoscopy is needed.

The use of ultrasound (US) in anesthesiology and emergency medicine has been rapidly increasing because of its safety and simplicity.^4^^,^^7^^,^^8^ Previous studies have found that difficult airway cases are associated with longer cutaneous-epiglottic distances (CED) as measured by US.^9^ Although this assessment provides seemingly good predictive results, there is limited data regarding the optimal cut-off point to make it reliable for routine clinical practice. This is primarily due to lack of technical standardization (i.e., how measurements are obtained) and small sample sizes in published studies.^10^ We, therefore, aimed to verify the predictive capability of US-measured CED in identifying patients who are potentially difficult to intubate under direct laryngoscopy (defined as Cormack-Lehane grades 3‒4). Secondarily, we evaluated potential correlations between CED, sex, and patient’s body mass index (BMI), and assessed for independent associations between CED and increased odds for difficult intubation.

## Materials and methods

The study was approved by the Research Ethics Committee of the Faculdade de Medicina de Botucatu – UNESP (protocol 4.961.886) in September 2021 and registered in the Brazilian Registry of Clinical Trials (U1111-1275-1120) in August 2022. This report followed the Standards for Reporting of Diagnostic Accuracy Studies protocol.^10^ Patients aged between 18 and 70 years who were scheduled for elective surgery under general anesthesia and tracheal intubation at the Centro Médico Campinas Foundation Hospital in Brazil were included. Patients with a BMI > 35 kg.m^-2^ and those with a previous history of difficult tracheal intubation and/or any difficult airway predictors (short thyromental/sternomental distance, reduced mouth opening, upper lip bite test, or other tests) were excluded. Patients in whom adequate US measurement was impossible due to technical reasons (e.g., prior radiotherapy or surgery to the neck, goiter, soft tissue masses), incorrectly recorded data, or those who had difficulty with bag-mask ventilation after induction of general anesthesia and/or required alternative means of tracheal intubation other than direct laryngoscopy were excluded.

**Figure 1:**
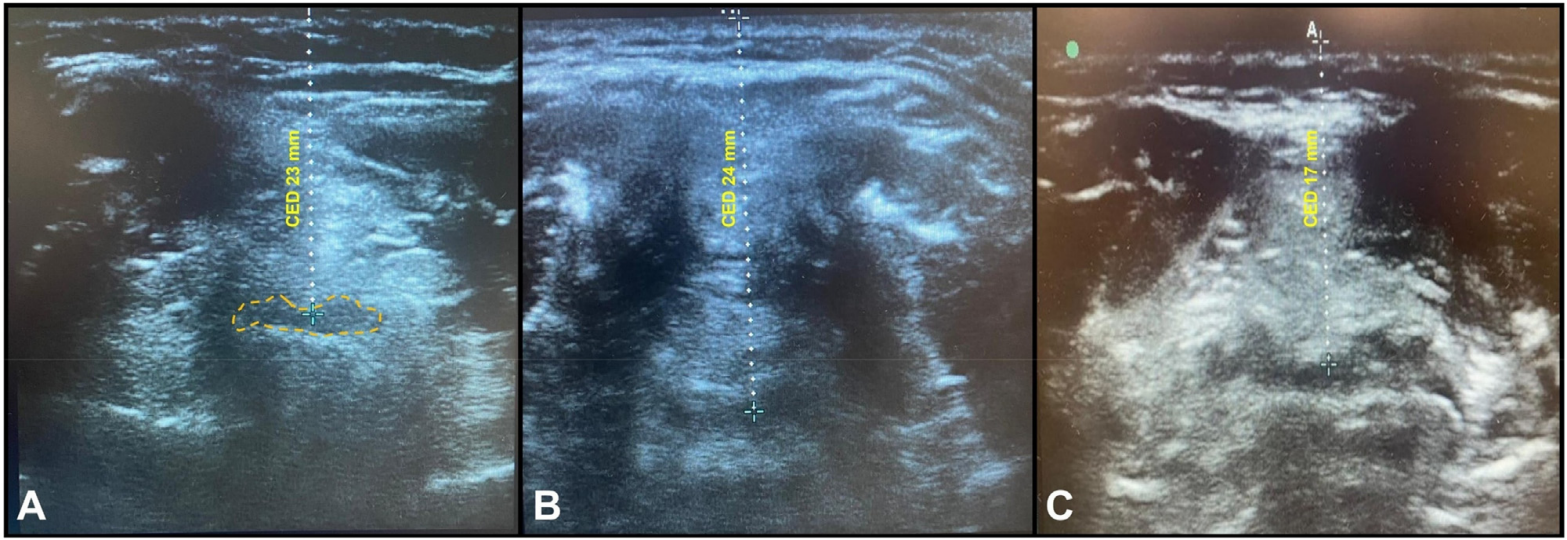
Airway ultrasound images and respective cutaneous-epiglottic distance (CED) measurements. The epiglottis is outlined (dashed orange line) in panel A.

All scans were performed by an expert airway ultrasonographer (LHC). CED was measured using a high-frequency (6–13 MHz) linear US transducer in the axial/transverse position, in the region surrounding the thyroid cartilage, as described by Kundra et al.^4^ The epiglottis was visualized as a hypoechoic semi-curvilinear structure in the midline with its anterior border delineated by the hyperechoic pre-epiglottic space and its posterior border by a bright air-mucosal interface corresponding to the air column in the airway. Once the image was obtained, a measurement line was drawn (with minimal pressure on the US probe) from the skin to the epiglottis (CED, in millimeters). All CED measurements were taken with the patients lying supine in the “sniffing” position. All US images were deemed adequate and CED measurements took an average of 180 seconds per patient. Figure 1 illustrates airway US images and their respective CED measurements.

All CED measurements took place immediately after induction of general anesthesia (GA) such that the patient's head was not repositioned between the measurement and tracheal intubation. Induction of GA was achieved with midazolam, fentanyl or remifentanil, propofol and rocuronium. Patients were kept hemodynamically stable, in an adequate depth of anesthesia, and ventilated under a facial mask with 100% oxygen before tracheal intubation. Direct laryngoscopy with appropriately sized Macintosh blades was performed by an experienced anesthesiologist after confirming the absence of muscular response (train-of-four of zero). The Cormack-Lehane classification was assessed in the first attempt as follows: Grade 1 = full view of the glottis; Grade 2 = partial view of the glottis and/or arytenoid cartilages visualized; Grade 3 = only the epiglottis visualized; and Grade 4 = neither the glottis nor the epiglottis visualized.

The same anesthesiologist performed up to three attempts at tracheal intubation under direct laryngoscopy. In exceptional circumstances of inability to intubate after the third attempt, a more experienced anesthesiologist, if available, was permitted one additional attempt. If, for any reason, tracheal intubation was not possible under direct laryngoscopy, alternative means such as videolaryngoscopy, flexible fiberoptic scope, optic stylet, and/or surgical airway kit were available at the discretion of the attending anesthesiologist.

### Statistical analysis

A descriptive analysis of the study population was performed, and the following data were analyzed: age, sex, weight, height, BMI, type of surgery, and number of attempts required for tracheal intubation.

To evaluate the clinical relevance of CED as a predictor of difficult tracheal intubation, a receiver operator characteristic (ROC) curve analysis was performed, and the sample size calculation considered a 25% prevalence of Cormack-Lehane grades 3‒4 in a mixed surgical population.^9^ For practical clinical discriminatory capability, we assumed that an area under the ROC curve (AUC-ROC) of at least 0.85 would be required to be proven by the evaluated method, and that 100 individuals would provide a width of 0.2 around this area, within a 95% confidence interval (95% CI).

Categorical variables were expressed as absolute and relative frequencies, and continuous variables were expressed as mean and standard deviation, as the Shapiro-Wilk test and histogram analysis showed a near-normal distribution.

The ROC curve was constructed by plotting the true positive rates (sensitivity) on the Y-axis and the false positive rates (1 – specificity) on the X-axis, and the optimal cut-off point was defined by the highest Youden index, in which the sum of sensitivity and specificity is maximized. The p-value for the AUC-ROC was evaluated under the null hypothesis of an area of 0.5, which denotes a 50/50 probability of either a true or false-positive result for detecting potentially difficult airways.

Secondary analyses were performed using the independent Student's *t*-test to compare the CED measurement in patients with Cormack-Lehane grades 1‒2 versus those with grades 3‒4. The relationship between CED and BMI was assessed using multiple linear regression, with sex as an independent covariate.

Binary logistic regression was also performed presenting the CED results in millimeters and the patients’ BMI as covariates for predicting Cormack-Lehane grades 3‒4 as a dichotomous outcome, since both covariates showed positive correlations.

A p-value < 0.05 was considered statistically significant.

## Results

This study included 100 patients whose assessments were conducted between November 2023 and May 2024. Table 1 presents the demographic data of the study population. The average age of the patients involved in the study was 47.6 ± 14.2 years, 54% were male, and the average BMI was 26.4 ± 3.7 kg.m^-2^. We identified 24 patients with Cormack-Lehane grades 3‒4 among the 100 participants. Tracheal intubation was performed on the first attempt in 86% of the cases, and all patients were successfully intubated using direct laryngoscopy.

**Table 1:** Demographic data, type of surgery, Cormack-Lehane classification, and frequency of tracheal intubation attempts.

Variable	Value
Age^a^	47.6 ± 14.2 years
Sex^b^	
Male	54 (54%)
Female	46 (46%)
Weight^a^	76.2 ± 14.6 kg
Height^a^	169.7 ± 9.3 cm
BMI	26.4 ± 3.7 kg.m^-2^
Cormack-Lehane grade^b^	
1 or 2	62 (72.1%)
3 or 4	24 (27.9%)
Frequency of intubation attempts^b^	
1	86 (86%)
2	8 (8%)
3	5 (5%)
4^c^	1 (1%)
Type of surgery^b^	
Abdominal	34 (34%)
Orthopedic	30 (30%)
Ear, nose, and throat	15 (15%)
Neurosurgery	12 (12%)
Urologic	3 (3%)
Thoracic	3 (3%)
Cardiac	3 (3%)

a Values expressed in mean ± standard deviation.

b Values expressed as absolute and relative frequencies.

c Three attempts by the same anesthesiologist with an additional attempt by a more experienced specialist.

**Figure 2:**
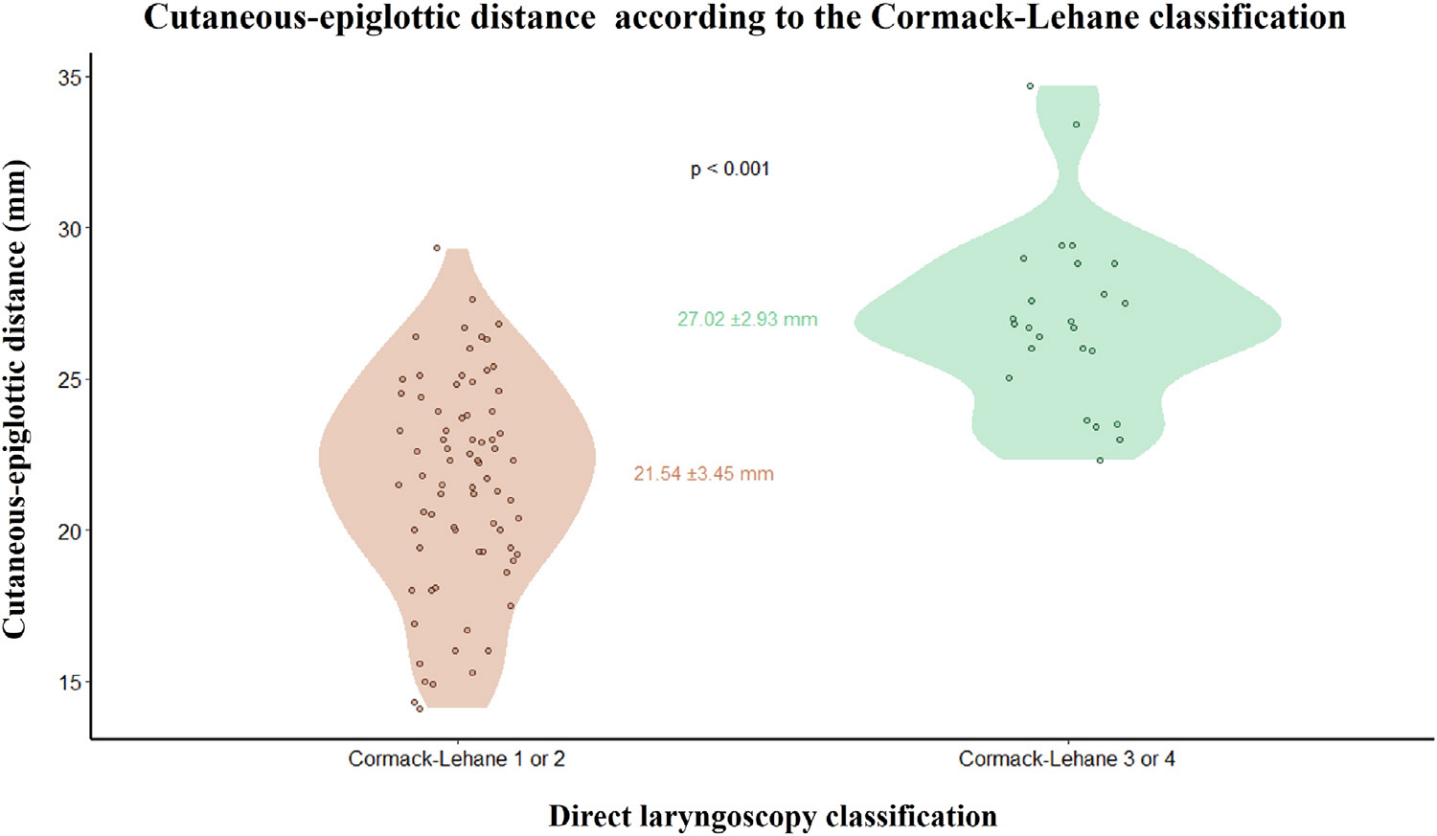
Relationship between the cutaneous-epiglottic distance and the Cormack-Lehane classification with respective means and standard deviations.

Figure 2 shows the distribution of patients according to the Cormack-Lehane classification and the respective mean CED values in a violin plot, indicating that patients with Cormack-Lehane grades 3‒4 presented longer distances than those with grades 1‒2.

The only patient who was intubated after the third attempt was a 68-year-old female with a BMI of 22.3 kg.m^-2^. In this case, the CED was 26.9 mm, and the Cormack-Lehane score was 4.

**Figure 3:**
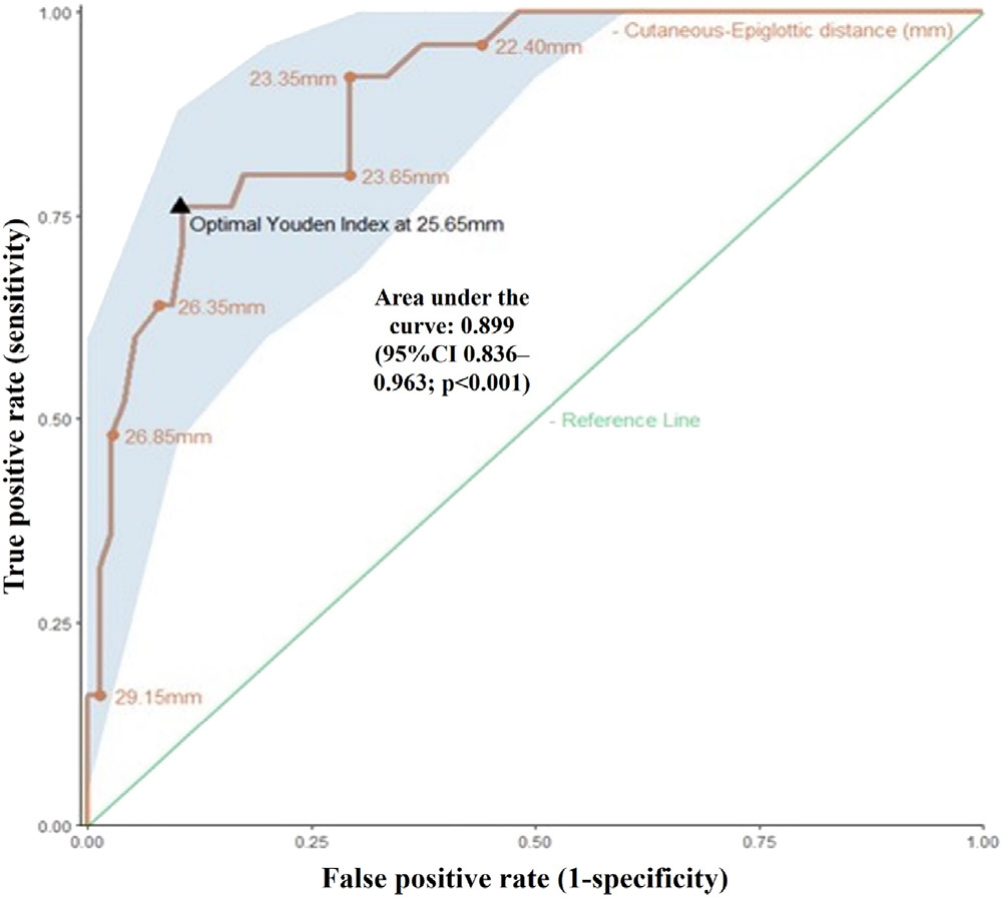
Receiver operator characteristics curve of the cutaneous-epiglottic distance for prediction of Cormack-Lehane grades 3 or 4. The cut-off value of 25.65 mm was identified using the Youden index (where the sum of the sensitivity and specificity is maximized) corresponding to an area under the curve of 0.899, indicating excellent performance of the CED in identifying patients with Cormack-Lehane 3 or 4. The accuracy of the CED was 76%, i.e., for every 100 patients, the CED was able to identify 76 patients with difficult laryngoscopy when measurement ≥ 25.65 mm. CI, Confidence Interval.

Discriminatory ROC curve analysis showed an AUC of 0.899 with a p-value of < 0.001 (null hypothesis of an AUC of 0.5), as shown in Figure 3. The maximum cut-off point determined by the Youden index was 25.6 mm, obtained using the best sensitivity and specificity values for CED. Values equal to or above this threshold showed a detection rate of 76 out of 100 patients with potentially difficult laryngoscopy, with false positives in 1 out of 10 individuals.

**Figure 4:**
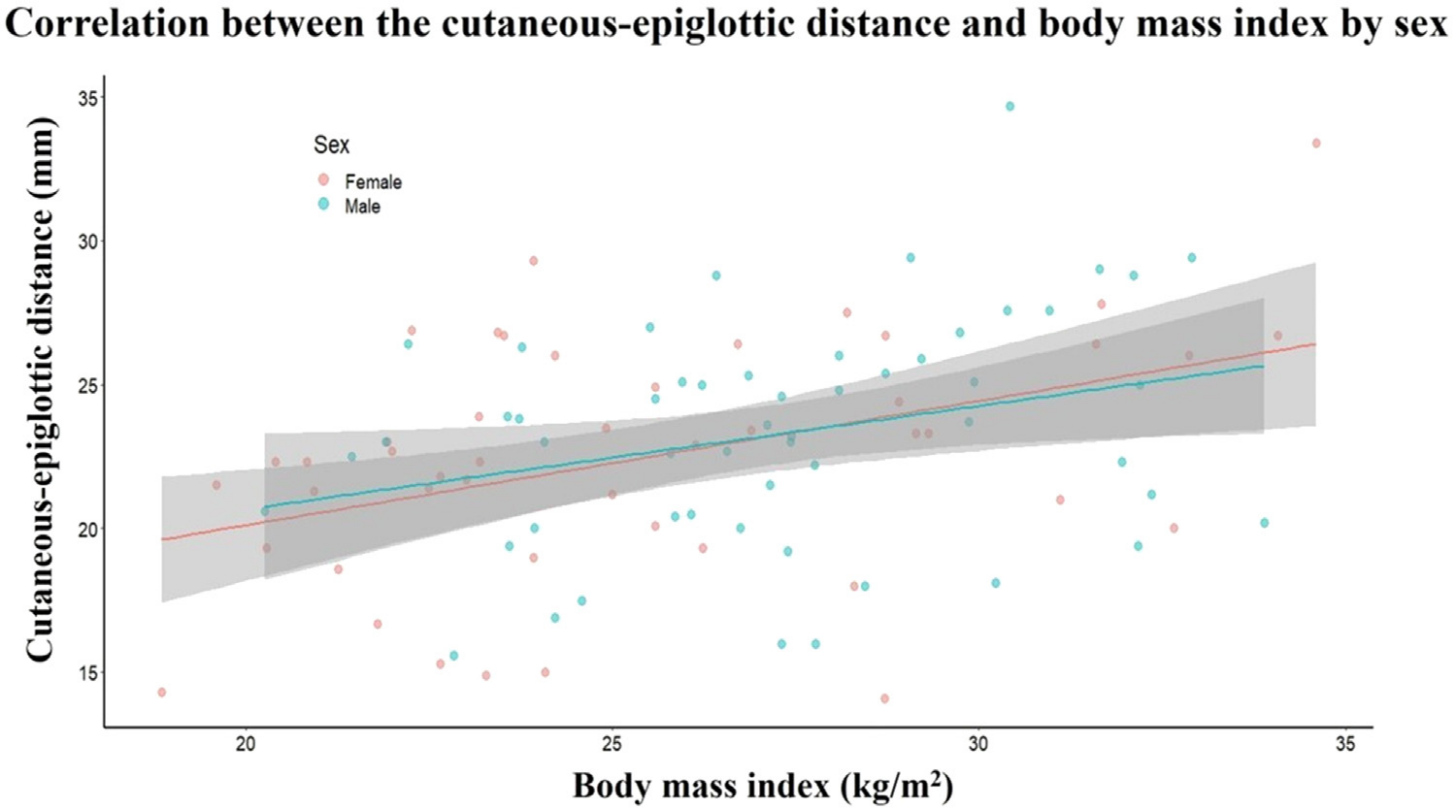
Scatter plot of cutaneous-epiglottic distance and body mass index by sex with respective linear regression lines, p-values, and confidence intervals.

Figure 4 shows that CED and BMI were positively correlated, and that this correlation was not significantly affected by sex. The binary logistic regression results in Table 2 demonstrate an independent increase in the odds ratio for difficult laryngoscopy with each additional point increase in CED and BMI. For CED specifically, a nearly 80% increase in the odds of Cormack-Lehane grades 3‒4 was observed for each millimeter increase in CED distance.

**Table 2:** Binary logistic regression for Cormack-Lehane grades 3 or 4 according to the cutaneous-epiglottic distance (mm) and the body mass index (kg.m^-2^).

Variable	Odds ratio (OR)	OR 95% Confidence Interval	p-value
Cutaneous-epiglottic distance	1.81	1.35–2.41	< 0.001
Body mass index	1.30	1.05–1.59	0.014

## Discussion

Technological advances and greater access to equipment and airway management techniques have contributed to reduce the risks associated with unanticipated difficult airways.^5^^,^^8^^,^^11^, ^12^, ^13^, ^14^ Accordingly, our findings suggest that US-measured CED may help predict Cormack-Lehane grades 3‒4 prior to direct laryngoscopy in adult patients without other classic predictors of difficult airway. Since the patients studied had no predictors of difficult airway, CED measurements were deliberately performed after induction of GA to mimic situations in which anesthesiologists may be surprised by an unanticipated difficult airway following anesthesia induction. Indeed, this study represents the daily life of anesthesiologists and can be easily reproduced using an innocuous method with a rapid learning curve. Importantly, the ROC curve analysis highlights CED’s excellent discriminatory capability for predicting Cormack-Lehane grades 3‒4 under direct laryngoscopy.

Assessment of the airway using anatomical predictors such as mouth opening, tongue thickness, and hyomental distance are confined to the observation of external features and have limited positive predictive values (8%‒20%). US can be useful for identifying potentially difficult airways by assessing the thickness of soft tissues and the inner aspects of the submandibular and neck areas.^1^^,^^15^, ^16^, ^17^, ^18^

Multiple US-guided measurements have recently gained relevance in the specialized literature, such as the distance from skin to the hyoid bone, the relationship between the pre-epiglottic space (distance from the skin to the epiglottis) and the sub-epiglottic space (from the epiglottis to the anterior commissure),^19^ evaluation of the angle formed between the glottis and the epiglottis, and the ratio between the hyomental distance with the head in neutral position vs. in extension,^20^ among others. It has also been noted that combining these measurements with the Mallampati score may further enhance predictive accuracy.^20^, ^21^, ^22^, ^23^, ^24^

In a prospective observational study, Falcetta et al. attempted to determine the correlation between the sonographic measurements of anterior cervical soft tissue thickness and Cormack-Lehane grade on direct laryngoscopy in 301 patients without predictors of difficult airway.^24^ Pre-epiglottic area of 5.04 cm^2^ (sensitivity = 85%; specificity = 88%) and mean CED of 25.4 mm (sensitivity = 82%; specificity = 91%) were considered good predictors for diagnosing patients with Cormack-Lehane grade ≥ 2b,^19^ the latter being similar to (and therefore corroborated by) the results (25.6 mm) found in the present investigation.

In a recent systematic review and meta-analysis comprising 10 studies and 1,810 patients focused on the ability of US to predict difficult laryngoscopy in patients without significant clinical/anatomical predictors, Carsetti et al. demonstrated high accuracy for CED in predicting difficult laryngoscopy with an AUC-ROC of 0.87 (95% CI 0.84–0.90).^24^ As expected, CED was higher in patients with higher Cormack-Lehane grades.^24^ Once again, our findings corroborate these results, with a CED AUC-ROC of 0.899 for detecting patients with Cormack-Lehane grades 3‒4. Interestingly, Carsetti et al. suggest that, despite its excellent sensitivity and specificity, CED is probably most useful when its result is negative, in which case the probability of an easy laryngoscopy is approximately 95%‒97%.^24^ This is due to the low prevalence (10%‒20%) of patients with difficult laryngoscopy in the studied populations. Conversely, a positive CED result suggests approximately 30%‒50% probability that the patient will indeed present a difficult airway.^24^

Although these (and several other) studies have documented CED as a predictor of difficult laryngoscopy, the ideal cut-off point and potential interactions with other factors such as sex and BMI remain undetermined.

CED showed a positive correlation with BMI, which was not influenced by sex, as adipose tissue accumulates in the submandibular region somewhat evenly in both men and women. Despite this positive correlation, any increase in CED or BMI was independently associated with greater odds of worse glottic visualization during laryngoscopy.^17^

Our analysis showed that a higher Youden index was obtained with a CED of 25.6 mm which represents the point of optimal trade-off between true and false positive results. Considering this cut-off point, a positive test (equal or higher value) would increase the baseline (*a priori*) chance of difficult laryngoscopy by 7.6 times (likelihood positive ratio). With an incidence rate of 27.9%, as observed in our sample, this translates into a positive predictive value of 74% and a negative predictive value of 91%.

Failure to recognize a problematic airway can be dangerous in clinical practice. Anesthesiologists should approach patients with positive test results cautiously, by using videolaryngoscopy, for example, as some of these cases will indeed present difficulties. Being overzealous in this setting may be prudent.^24^ Therefore, determining the ideal cut-off point is not as straightforward as univariate comparisons of CED and other population variables, even though the observed AUC-ROC was clinically significant.^9^

Owing to the inherent risks associated with difficult airways and the potential for catastrophic results, one might argue that it is better to increase the detection rate, even at the expense of a higher false-positive rate. For example, reducing the cut-off point to 23.4 mm would detect 88% of patients with Cormack-Lehane grades 3‒4, with a negative predictive value of 94%. However, this would also mean that approximately 30% of the patients with lower visualization grades would have values equal to or higher than this threshold, with an unavoidable reduction in the positive predictive value to 54%.

The cut-off values for CED considered predictive of difficult laryngoscopy and found in previous studies^9^^,^^20^, ^21^, ^22^ range from 25.4 mm to 27.5 mm, with an AUC-ROC of 0.693‒0.903, which may be explained in part by differences in ethnicity and other population characteristics. Our results not only corroborate those findings but further extend the current knowledge by showing that with each millimeter increase in CED, the odds of Cormack-Lehane grade 3‒4 on direct laryngoscopy increase by approximately 80%.

This study has limitations. This was a single-center investigation which may limit the external validity of our findings. Additionally, we did not include patients with known markers of difficult airway (e.g., limited mouth opening or restricted neck extension) which could have increased the incidence of Cormack-Lehane grades 3‒4. Future larger multi-center studies are, therefore, warranted to confirm our findings in a broader population. Similarly, CED measurements took place after induction of anesthesia and muscle relaxation which may not reflect preoperative conditions. Furthermore, there is a tendency for videolaryngoscopy to become the standard technique for tracheal intubation, particularly in patients with suspected difficult airways, and the classic predictors of difficulty in direct laryngoscopy may not apply in this context. Finally, due to the exploratory nature of our secondary objectives and the absence of specific sample size calculations or correction methods used for multiple comparisons, our results should be interpreted cautiously. Type II errors may occur with an unspecified probability in situations where there are no significant differences, and higher chances of type I errors are expected due to the family-wise error rate.

The incorporation of CED into institutional protocols requires scientific dissemination and adequate clinician training.^25^ Point-of-care ultrasound has become ubiquitous in anesthesiology such that CED measurements could be seamlessly integrated into our daily practice. Given its characteristics (simple, reproducible, noninvasive) and documented advantages (rapid learning curve and excellent discriminatory capability), US-based CED has a great potential to be routinely incorporated as an additional tool (besides the classic anatomical tests/landmarks) to predict difficult airways. However, further studies are warranted to determine CED’s validity and reliability as a predicting tool in a variety of settings (e.g., spontaneous ventilation, poor cervical spine mobility) and patient populations (pediatric, obstetric, and obese patients, as well as those with known anatomical predictors of difficult airway).

## Conclusion

Ultrasound-measured CED demonstrates strong discriminatory ability in distinguishing between easy (Cormack-Lehane grades 1‒2) and difficult (grades 3‒4) laryngoscopy, highlighting its potential role in preoperative airway assessment. However, further research is required to validate its accuracy in high-risk populations and to assess its reliability in pre-induction settings.

## Ethics approval

This investigation was approved by the UNESP – Faculdade de Medicina de Botucatu Research Ethics Committee (Protocol 4.961.886) and was registered in the Brazilian Registry of Clinical Trials (U1111-1275-1120). Written patient consent was obtained as appropriate.

## Prior presentations

None.

## Authors’ contributions

Luis Henrique Cangiani: Conceptualization; methodology; formal analysis; investigation; data curation; project administration; writing-original draft; writing-review, edit and approval of final manuscript.

Rodrigo Leal Alves: Conceptualization; methodology; investigation; data curation; project administration; writing-original draft; writing-review, edit and approval of final manuscript

Glenio B. Mizubuti: Formal analysis; writing-original draft; writing-review, edit and approval of final manuscript.

Rodrigo Moreira e Lima: Writing-review, edit and approval of final manuscript.

Lais Helena Navarro e Lima: Conceptualization; methodology; formal analysis; investigation; data curation; project administration; writing-original draft; writing-review, edit and approval of final manuscript.

All authors reviewed and edited the manuscript with important intellectual contributions and approved the final version for submission for publication.

All authors conceived, drafted and critically revised the manuscript, and approved the final version submitted for publication in the Brazilian Journal of Anesthesiology.

## Funding

This work did not receive any specific grant from funding agencies in the public, commercial, or not-for-profit sectors. Support was provided solely by departmental and institutional resources.

## Conflicts of interest

The authors declare no conflicts of interest.
